# Bigger mothers are better mothers: disentangling size-related prenatal and postnatal maternal effects

**DOI:** 10.1098/rspb.2013.1225

**Published:** 2013-09-07

**Authors:** Sandra Steiger

**Affiliations:** 1 Institute of Experimental Ecology, University of Ulm, Albert-Einstein-Allee 11, 89069 Ulm, Germany; 2 Institute of Biology 1, University of Freiburg, Hauptstraße 1, 79104 Freiburg, Germany

**Keywords:** maternal effects, body size, egg size, parental care, burying beetle, *Nicrophorus*

## Abstract

Despite a vast literature on the factors controlling adult size, few studies have investigated how maternal size affects offspring size independent of direct genetic effects, thereby separating prenatal from postnatal influences. I used a novel experimental design that combined a cross-fostering approach with phenotypic manipulation of maternal body size that allowed me to disentangle prenatal and postnatal maternal effects. Using the burying beetle *Nicrophorus vespilloides* as model organism, I found that a mother's body size affected egg size as well as the quality of postnatal maternal care, with larger mothers producing larger eggs and raising larger offspring than smaller females. However, with respect to the relative importance of prenatal and postnatal maternal effects on offspring growth, only the postnatal effects were important in determining offspring body size. Thus, prenatal effects can be offset by the quality of postnatal maternal care. This finding has implications for the coevolution of prenatal and postnatal maternal effects as they arise as a consequence of maternal body size. In general, my study provides evidence that there can be transgenerational phenotypic plasticity, with maternal size determining offspring size leading to a resemblance between mothers and their offspring above and beyond any direct genetic effects.

## 1. Introduction

Body size is an important life-history trait that has received considerable attention in the last decades [1–6]. In general, variation in body size reflects the interaction of additive genetic components with various environmental factors, such as temperature, food availability or maternal effects, like the quality of parental care received. Although maternal effects are an environmental source of variation for offspring phenotype, they can have a genetic basis when the variation in the quality of the environment provided by the mother reflects genetic differences among mothers (i.e. indirect genetic effects) [7]. Whether body size is a highly heritable trait or largely determined by environmental experiences, strongly depends on the animal species in focus, but in many organisms maternal effects have been identified as a major contributor to variation in offspring body size: offspring size can be influenced by propagule resource allocation, oviposition strategies (e.g. when, where and how many eggs are laid), postnatal brood size regulation, the degree of maternal care or mate choice [8,9]. Also, maternal traits such as age during reproduction or body size can provide specific environments that give rise to maternal effects and drive meaningful variation in offspring size [10–12]. For example, larger mothers might be better in defending resources, produce larger eggs or be able to provide more food to offspring. As a result, maternal variation in body size can be environmentally transmitted across multiple generations and—because body size often influences fecundity and survival—affect the fitness of individuals across multiple generations [13].

The evolution of body size is of substantial interest, because there is tremendous variation in these traits at all taxonomic levels. However, to obtain a complete picture of the evolutionary responses to selection on body size, we not only need an understanding of the direct genetic effects on body size, but also of the maternal effects, as these can accelerate or limit the response to selection [7,14]. Studies were able to show—via half sib breeding designs or cross-fostering experiments—that maternal body size can influence offspring size independent of direct genetic effects. For example, in the dung beetle *Ontophagus taurus* maternal body size exerts a strong influence on the weight of brood masses produced, accounting for 22 per cent of the non-genetic variance in offspring body size [15]. However, remarkably little is known about whether the effect of maternal body size on offspring size arises predominantly via prenatal (e.g. egg size) or via postnatal maternal effects (e.g. parental care). In general, disentangling prenatal and postnatal effects is important for our understanding of the evolution of size-related maternal effects, as these two temporally separated mechanisms might evolve independently or as co-adapted traits [16]. For example, postnatal maternal effects may compensate for any negative influence of prenatal maternal effects or vice versa: If smaller females are morphologically constrained to lay smaller eggs, they might counter this with better postnatal care. Or, if large females are more fecund and thereby incidentally reduce the quality of their offspring's environment by increasing sibling competition, they might increase parental investment to offset the negative effects of sibling competition [17].

In the current study, I specifically examined size-related maternal effects, thereby disentangling prenatal from postnatal maternal effects and analysing their relative contribution to offspring growth. I used a cross-fostering design combined with non-genetic manipulation of maternal body size that allowed me to control for any confounding genetic effects on offspring size. I conducted my study in burying beetles, *Nicrophorus vespilloides*, as they provide elaborate post-hatching parental care that involves direct provisioning of food to offspring by regurgitation [18,19]. Hence, in this model species there is potential for both size-related prenatal, as well as postnatal maternal effects. Burying beetles reproduce on dead vertebrates that serve as the sole food source for their offspring [19]. Body size is a very plastic trait that strongly depends on the relationship between carcass size and brood size in this group of beetles [20–22]. Parents have—at least to some extent—control over the number and size of the offspring they produce because they regulate offspring number at two points during reproduction. First, females adjust the number of eggs laid to carcass size [23] and second, both parents regulate brood size through filial cannibalism [24]. Body size significantly affects individual fitness, as larger beetles usually are victorious over smaller individuals in fights for possession of carcasses suitable for reproduction [25]. In addition, large individuals are more effective in guarding the brood. While the advantage of large body size in contests is clear, it is unknown whether variation in maternal body size induces any phenotypic variation in offspring traits.

Body size of burying beetles can be easily manipulated by removing final instar larvae from the carcass at different points of time, thereby preventing any further feeding, which result in different larval masses. After their removal from the carcass, the larvae pupate and body size after eclosion strongly correlates with larval mass [20]. Larvae of low mass also pupate under natural condition as, for example, when there are many offspring on a small carcass and sibling competition for food is high, or when eggs are laid asynchronously and some larvae arrive late at the carcass. The manipulation allowed me to generate full siblings with large non-genetic differences in body sizes. In this study, I generated small and large females that served as mothers. In the first experiment, I analysed potential size-related prenatal maternal effects, in a second experiment, I examined size-related postnatal maternal effects, keeping all prenatal effects constant across treatment groups. In the final experiment, I analysed the relative contribution of prenatal and postnatal maternal effects to final offspring mass by cross-fostering offspring produced by small or large females with small or large carers. I expected that maternal body size gives rise to both, prenatal and postnatal maternal effects, but that the size-related postnatal effects are much more pronounced due to the burying beetles’ elaborate form of post-hatching parental care.

## 2. Material and methods

### (a) Collection and maintenance of beetles

Experimental animals used in the study were F_2_ offspring of *N. vespilloides* beetles trapped in carrion-baited pitfall traps in a deciduous forest near Freiburg, Germany. The breeding method applied allowed me to generate beetles free of nematodes and mites that are typically found on field-caught beetles (see [26] for detailed a description of the breeding protocol). Consequently, I could avoid variation in parents’ performance due to parasitism. After eclosion of the adults until the beginning of the experiments, beetles were maintained in groups of up to five same-sex siblings in small transparent plastic containers (10 × 10 × 8 cm) at 20°C on a 16 L : 8 D cycle and provided with decapitated mealworms twice a week. All experimental females used were sexually mature and 20–40 days of age.

### (b) Breeding protocol for generating siblings of different body sizes

To obtain *N. vespilloides* beetles that differed greatly in body size, pairs of beetles (F_1_ offspring) were provided with 20-g mouse carcasses suitable for reproduction. After hatching, larvae were checked daily to determine their developmental stage. As soon as they had reached their third and final instar, the mass of each larva within a brood was measured daily. Once they had achieved a mass of 90–120 mg, about half of the brood was removed from the carcass and transferred to a new container filled with moist peat for pupation. The other half remained with their parents until the larvae left the carcass for pupation on their own. These larvae were weighed and transferred to a new box as well. This procedure allowed me to generate full siblings from 30 different families with a substantial difference in body size. After eclosion, the small females had a mean (±s.d.) pronotum width of 3.97 mm (±0.21) and the large females, 5.54 mm (±0.23) (linear mixed effects restricted estimate maximum-likelihood (REML) model with family as a random effect: *F*_1,71_ = 1335.25, *p* < 0.0001). The size range was within the range of offspring sizes produced by parents in the laboratory and in the field (S.S. 2003, unpublished data). To avoid any diet-related differences, females of both size groups were provided with food ad libitum after eclosion.

### (c) Prenatal maternal effects: effect of adult body size on clutch size, egg size, larval hatching weight and hatching pattern

In my first experiment, I examined size-related prenatal maternal effects in the context of two different food supply treatments (low and high). To this end, I measured clutch size, egg size, larval hatching weight, initiation of larval hatching, hatching spread and egg-laying speed. These parameters all reflect a mother's prenatal resource allocation and have consequences for offspring phenotype. Although egg size and hatching weight have direct effects on offspring size, the other variables have indirect effects, as they inevitably influence the post-hatching competitive environment for siblings and food availability [27]. Two different food source conditions were chosen, because it is known that carcass size as well as nutrition prior to reproduction influence clutch size [23,28]. *Nicrophorus vespilloides* females provided with a carcass smaller than 10 g do not lay the maximum number of eggs they are physiologically able to produce but adjust clutch size to the size of the carcass. Only when provided with a carcass larger than 10 g, do they appear to lay the maximum number of eggs, as clutch size does not increase any further with increased carcass size, even though the carcass can support more offspring [23]. Hence, the low food supply treatment served to test whether female body size influenced decisions concerning clutch size regulation, whereas the higher food treatment was intended to determine whether there are size-related constraints on the maximum number of eggs females can lay. Because a carrion diet prior to reproduction positively influences clutch size [28], I added this treatment to obtain a more precise estimate of the maximum egg number females are able to produce. Therefore, females from both size groups (*n* = 40 for each) were either provided with a carrion diet ad libitum (small pieces of mouse carrion unsuitable for reproduction) for 10 days prior to reproduction, or they were fed with mealworms ad libitum. Females in the first group were then provided with 20-g (±0.2) mice (high food supply treatment), whereas those in the latter were provided with 5-g (±0.2) mice (low food supply treatment).

The general procedure for all females across treatment groups was the following: females of both size groups were left with a non-sibling male for 24 h to ensure that they received enough sperm for fertilization. Afterwards, individual females were weighed and then transferred to plastic boxes (10 × 10 × 6.5 cm) containing a freshly killed mouse on top of moist peat that filled about two-thirds of the box. These boxes were checked after 4 h and at least every 12 h thereafter. Once the carcass had been interred, females were transferred to a dark environmental chamber at 20°C to simulate underground conditions. After about 48 h, and if required, after another 48 h, females and their carcasses were carefully transferred to new plastic containers under red light and returned to the dark. The old boxes were searched for eggs. I stored the eggs in Petri dishes lined with moist filter paper at 20°C and checked them every 6 h until the first larva was observed. At that time, I transferred the females again to retrieve any additional eggs. These eggs were also transferred to Petri dishes lined with moist filter paper. In accordance with other studies, clutch size was defined as the total number of eggs laid until a female's first larva hatched, because females cease ovipositing before their first larvae reach the carcass [29].

Prior to hatching, I measured the size of the eggs. To avoid variation in egg size due to differences in development time, I determined egg size as soon as the mandibles and claws of the embryo were visible within an egg. I randomly selected five eggs from each female and measured their length and width using a stereomicroscope equipped with an ocular micrometer. The eggs were then returned to their respective Petri dish. The measurements were used to calculate a prolate spheroid volume *V* for each egg using the equation *V* = (1/6)*πw*^2^*L*, where *w* is the width and *L* the length of the egg [30].

I checked the Petri dishes every 6 h until all larvae had hatched, each time noting the number of larvae that had hatched from each brood and measuring the weight of the newly hatched larvae. To characterize the hatching pattern, I determined the hatching spread which is simply the time elapsed between the hatching of the first and the last larva from a clutch laid by a given female [31]. The quotient of hatching spread and clutch size gave me an estimate of egg-laying speed.

To examine the effects of maternal body size and food supply on prenatal resource allocation, I used linear mixed effects REML models in SPPS v. 20. I included body size (large or small) and food supply (low or high) as fixed factors, family from which the focal female originated as a random effect, and clutch size, mean egg size per brood, mean larval birth weight per brood, initiation of larval hatching, hatching spread and egg-laying speed as dependent variables. Females that laid unfertilized eggs or no eggs at all were not included in the analysis.

### (d) Postnatal maternal effects: effect of adult body size on offspring growth

This experiment was designed to examine the effect of size-related postnatal effects on offspring growth independent of size-associated prenatal effects. To that end, I standardized egg number and hatching pattern by providing the small and large females (each *n* = 18) from the low food supply treatment described above with 24 larvae to rear. This number was chosen as it corresponds to the average clutch size produced by small and large females on a 5-g carcass. To simulate hatching spread, the 24 larvae were not provided at once, but six larvae were added to each mother every 6 h, starting at the time the mother's first larva had hatched. The origin of larvae (originating from small or large females) was randomized by pooling all newly hatched larvae from small and large females in one Petri dish every 6 h, and then randomly distributing them among the females. This procedure ensured that each mother received larvae of mixed parentage with a similar average weight. Larval weight gain is highly dependent on the food availability, which in turn might depend on the pre-hatching quality of carcass conservation by the mother as well as on her personal food demand. Therefore, I carefully removed the female and the carcass for a short period of time and measured their weights before I added the first larvae. Broods were checked twice a day and when the carcass had been entirely consumed, the larvae were recovered from the peat and individually weighed. To assess the effect of maternal body size on offspring growth, I used mixed effects REML models, including body size (large or small) as the fixed factor, family as a random factor and the number and mean weight of larvae per brood at the end of the breeding phase as dependent variables. In addition, I analysed the effect of maternal body size on the weight loss of the carcass and the weight gain of the respective female from the time at which the carcass was made available to the time at which the first larvae hatched using a MANOVA. In four cases, there were insufficient larvae to provide to females, and in one case, the female killed the entire brood. These females were excluded from the analyses.

### (e) Cross-fostering experiment to evaluate prenatal and postnatal size-related maternal contributions to offspring body size

To determine the relative contribution of hatching weight (prenatal maternal effect) and quality of postnatal maternal care (postnatal maternal effect) to final offspring weight, I cross-fostered offspring produced by small or large females with small or large carers of the same age.

Females of both size groups were paired with males for 24 h and then provided with a 5-g (±0.2) mouse carcass. Forty-eight hours later, I transferred the mother along with the carcass to a new container. The old containers were checked for newly hatched larvae at 6-h intervals. As in the previous experiment, each female was provided with six larvae every 6 h until she had been provided with a total of 24 larvae, starting at the time the mother's first larva had hatched. Small females either received freshly hatched larvae hatching from eggs laid by small females or larvae from eggs laid by large females, and the same treatments replicated for large females. To ensure the timing of cross-fostered broods, multiple females were bred simultaneously. However, because of individual variation in the initiation of oviposition and to ensure that throughout the experiment I had enough newly hatched larvae derived from females of both size groups, I established many more females to serve as donor mothers (*n* = 100) than as carers (*n* = 39). Broods were checked twice a day and at the end of the parental period, when the carcass was entirely consumed, the larvae were recovered from the peat and individually weighed. I used two ANCOVAs to evaluate the relative importance of prenatal and postnatal size-related maternal effects, including body size of the donor mother (prenatal) and body size of the carer (postnatal) as fixed factors. In the first ANCOVA, the number of larvae per brood at the end of the breeding phase was added as dependent variables and mean weight of the larvae per brood as covariate. In the second ANCOVA, the mean weight was entered as the dependent variable and the number of larvae as covariate. This procedure was chosen, as many studies before have found a strong correlation between offspring number and size [20,24,32].

## 3. Results

### (a) Size-related prenatal maternal effects

**Figure 1.**
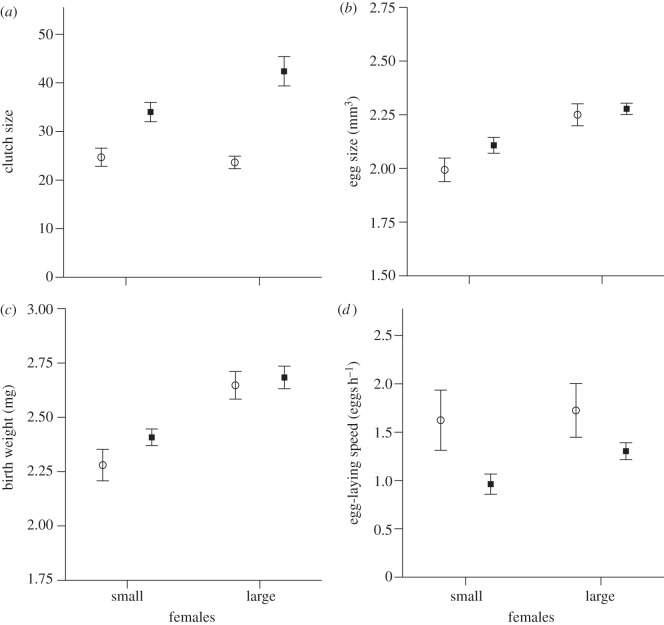
Body size-related prenatal maternal effects. Females were either subjected to a low food supply treatment (open circles) or high food supply treatment (filled squares). (*a*) Clutch size, (*b*) egg size, (*c*) larval birth weight and (*d*) egg-laying speed of small and large females. Data shown as means ± s.e.

Body size and food supply had an effect on the measured parameters for prenatal maternal effects. The size of the mother did not affect clutch size, but the food supply had an effect, with females from the high food supply treatment laying more eggs than females from lower food supply treatment (table 1 and figure 1*a*). However, the mixed model showed a significant interaction effect between body size and food supply in their effects on clutch size (table 1). Whereas in the low food size treatment, there was no effect of body size on clutch size, larger females laid more eggs than smaller ones in the high food supply treatment (figure 1*a*). Body size also had a strong effect on egg size and larval birth weight, with larger females producing larger eggs and heavier newborn offspring (table 1 and figure 1*b,c*). In contrast, food supply had no significant influence on egg size and larval birth weight, and there was no significant interaction between body size and food supply treatment on these reproductive parameters (table 1 and figure 1*b,c*). Not surprisingly, there was a strong positive correlation between larval birth weight and egg size (*n* = 63, *r* = 0.7, *p* < 0.001). There was no correlation between egg size and number within treatments, nor across all treatment groups pooled, suggesting that there is no trade-off between size and number of offspring at the egg stage (small females, low food supply: *n* = 15, *r* = 0.15, *p* = 0.58; small females, high food supply: *n* = 13, *r* = 0.19, *p* = 0.54; large females, low food supply: *n* = 18, *r* = 0.35, *p* = 0.16; large females, high food supply: *n* = 18, *r* = 0.14, *p* = 0.57; for all treatment groups together: *n* = 64, *r* = 0.24, *p* = 0.06). Body size did not influence the initiation of larval hatching or hatching spread, but larvae produced by mothers in the high food supply treatment hatched earlier, but over a longer time period than those in the low food supply treatment (table 1). There was no significant interaction between the size of the mother and her food supply with respect to the initiation or duration of hatching (table 1). Females provided with a small carcass laid their eggs at a higher rate than those provided with a large carcass (table 1 and figure 1*d*). Within the low food treatment, body size did not influence egg-laying speed (*F*_1,34_ = 0.34, *p* = 0.57), but within the high food supply treatment larger females laid more eggs per hour than smaller ones (*F*_1,30_ = 6.27, *p* = 0.02).

**Table 1.** Results of the mixed effects REML model with maternal family included as a random effect, showing the fixed effects of body size, food supply and their interactions on measurements of prenatal maternal effects. Bold values are statistically significant.

parameters	body size	food supply	body size × food supply
clutch size	*F*_1,59_ = 3.10	***F*_1,55_** **= 42.29**	***F*_1,55__=_ 4.94**
	*p* = 0.08	***p* < 0.001**	***p* = 0.03**
egg size	***F*_1,54_ = 23.36**	*F*_1,51_ = 2.92	*F*_1,52_ = 0.95
	***p* > 0.001**	*p* = 0.09	*p* = 0.33
larval birth weight	***F*_1,54_ = 30.81**	*F*_1,51_ = 2.18	*F*_2,52_ = 0.50
	***p* > 0.001**	*p* = 0.15	*p* = 0.48
start of hatching	*F*_1,59_ = 0.11	***F*_1,56_ = 11.61**	*F*_1,56_ < 0.001
	*p* = 0.75	***p* < 0.001**	*p* = 0.99
hatching spread	*F*_1,51_ = 1.21	***F*_1,48_ = 35.69**	*F*_1,49_ = 0.33
	*p* = 0.28	***p* < 0.001**	*p* = 0.57
egg-laying speed	*F*_1,55_ = 1.09	***F*_1,55_ = 32.68**	*F*_1,55_ = 0.32
	*p* = 0.30	***p* = 0.013**	*p* = 0.57

### (b) Size-related postnatal maternal effects

**Figure 2.**
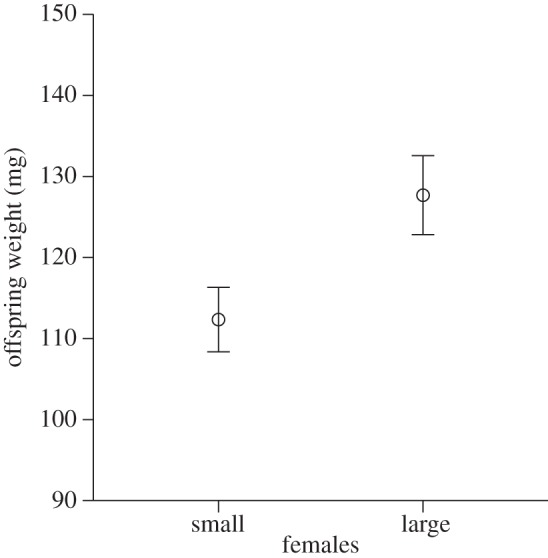
Body size-related postnatal maternal effects: mass of offspring raised by small or large mothers. Data shown as means ± s.e.

Up until the time of larval hatching, carcasses tended by large mothers lost relatively more weight (mean ± s.e.: 8.79% ± 1.60) than carcasses tended by small females (4.28% ± 1.40; *F*_1,33_ = 4.23, *p* < 0.05). This was likely caused by differences in food intake, as large females gained absolutely (45.39 mg ± 4.34), as well as relatively (16.69% ± 1.77), more weight than small females (absolute: 10.71 mg ± 2.07, *F*_1,33_ = 50.01, *p* < 0.001, relative: 9.70% ± 1.99, *F*_1,33_ = 6.91, *p* < 0.013). Body size had no effect on the number of larvae raised per brood (large females: 10.81 ± 0.65, small females: 10.40 ± 0.52, *F*_1,21_ = 0.10, *p* = 0.76), but even though carcasses of large mothers had lost more weight at the time the larvae hatched, large females raised significantly heavier larvae than small females (*F*_1,21_ = 5.86, *p* = 0.02; figure 2), suggesting that adult body size influences the quality of post-hatching maternal care. On 5-g carcasses, offspring increased their weight from an average of 2.5 (±0.3) mg at hatching to an average of 127.7 (±4.9) mg at dispersal when reared by large mothers, and to an average of 112.3 (±4.0) mg when reared by small mothers.

### (c) Combined prenatal and postnatal maternal effects related to adult body size

**Figure 3.**
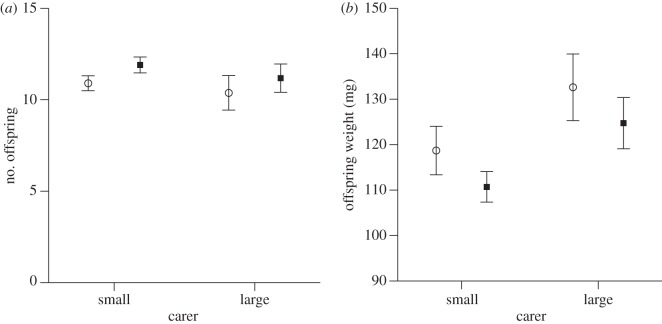
Combined prenatal and postnatal effects: (*a*) number and (*b*) mass of offspring deriving from small or large donor females and raised by small (open circles) or large (filled squares) carer females. Data shown as means ± s.e.

Neither the size of the donor mother (*F*_1,34_ = 0.11, *p* = 0.75) nor the size of the carer (*F*_1,34_ = 2.7, *p* = 0.11), affected the number of larvae reared per brood (figure 3*a*). Larval body mass at dispersal did not depend on the size of the donor mother (*F*_1,34_ = 0.33, *p* = 0.57), but was significantly affected by the size of the carer (*F*_1,34_ = 8.50, *p* = 0.006), with larger mothers rearing larger offspring. This suggests that larval hatching weight contributes relatively little to the final larval weight, but that the main factor determining offspring size is the nature of post-hatching maternal care. As in many studies before, the number of larvae reared per brood significantly affected their weight (*F*_1,34_ = 59.96, *p* < 0.001).

There was no significant interaction effect between donor and carer treatments on offspring weight (*F*_1,34_ < 0.031, *p* = 0.86), indicating that there are no obvious co-adaptations between size-related prenatal and postnatal maternal effects (cf. [16]). This is also reflected by the fact that the average larval body mass in those cases in which the size of the donor and carer matched (121.21 mg ± 3.86) was nearly identical to the average larval weight in the previous experiment (120.25 mg ± 3.42), in which females of both size classes received larvae of mixed origin (*F*_1,51_ = 0.09, *p* = 0.76).

## 4. Discussion

The main objective of this study was to investigate the non-genetic effects of maternal body size on progeny phenotype, thereby disentangling prenatal from postnatal maternal effects. To that end, I used a design that combined an environmental manipulation of adult body size with a cross-fostering approach. I found that the mother's body size affected both prenatal and postnatal offspring traits. However, when looking at the combined prenatal and postnatal maternal effects and analysing their relative contributions to the final offspring phenotype, it became apparent that only the postnatal contribution resulted in a significant modification of final offspring phenotype.

The strongest prenatal maternal effect found was that on egg size: irrespective of resource size, larger females produced larger eggs that resulted in a higher larval hatching weight. This is not an unusual finding as in many organisms maternal body size determines egg size and therefore the size of hatchlings [33–36]. A proximate explanation for this relationship might be morphological constraints, as the available body space might determine the overall egg volume that can be carried, or the size of the maternal organ through which eggs pass during oviposition might impose constraints [37]. Alternatively, there might be metabolic constraints such that the resource transport rate from the mother to the egg (e.g. vitellogenin uptake) is limited by body size (‘stream-limitation hypothesis’; [38]). Final egg maturation in burying beetles does not take place before a carcass is found [39,40]. As carcass quality deteriorates steadily over time, females should be selected to initiate oviposition as quickly as possible. However, the resource transport rate for ovarian development might be constrained by body size, leading to smaller eggs in females of smaller body size when the onset of oviposition between large and small females does not differ (which was the case in the present study).

Large females produced more eggs than small ones, but only in the high food treatment. It is not uncommon for fecundity to scale positively with maternal body size [41], and the underlying mechanism might be again a morphological constraint arising from available body space. However, burying beetles are known to lay their eggs asynchronously [31,42], and they are also able to reproduce a replacement clutch when the first brood is lost [29]. Thus, body volume is probably not a major constraint because females could potentially manufacture new mature eggs after oviposition has already commenced. A more likely constraint is the time span from the first to the last egg laid, which determines the overall hatching spread (see also [23]). Smiseth & Morgan [43] showed that offspring survival was lower in highly asynchronous broods (hatching spread: 48 h) than in synchronous (hatching spread: 0 h) or moderately asynchronous broods (hatching spread: 24 h). In this study, the hatching spread on a large carcass was about 40 h for small females and 35 h for large females. Hence, large females needed less time per egg to produce and lay than small females. Therefore, the rate of egg laying may be the limiting factor for the size of a clutch in burying beetles.

Female body size affected not only the size of eggs, but the quality or quantity of postnatal maternal care provided by the mother. Even when prenatal factors were held constant, large mothers raised larvae of higher mass than small ones. Although my results provide no information about the mechanism by which post-hatching parental care is influenced by maternal body size, it is likely that it involves differences in the efficiency of food allocation to the offspring. Females have to ingest, partly digest and regurgitate carrion. The amount of food that can be ingested and processed by proteolytic enzymes in a specific time may simply depend on a carer's body size. Food provisioning can be directly observed in burying beetles [44,45], and thus it would be interesting to determine whether the overall time spent in mouth-to-mouth contact with the larvae depends on the mother's body size. However, the results of such a study would have to be interpreted with caution as the provisioning time might not reliably reflect the actual amount of food allocated to the offspring. Body size has been shown to affect food provisioning in other arthropods as well. During reproduction, female dung beetles remove portions of dung from dung pads as nutrition for the developing young. Hunt & Simmons [46] found that female body size influences the quantity of dung provided, which in turn determines offspring body size.

In this study, final offspring size was independent of egg volume and, thus, hatching weight, but were affected by differences in post-hatching care. These results corroborates Ricklefs’ [47] contention based on findings in starlings that postnatal parental care has a much stronger effect on offspring growth than egg size. Although in other species with post-hatching parental care, egg size has been found to have an independent effect on offspring fitness (see references in [48]), post-hatching care often seems to mask or modify these effects [12]. In the cooperatively breeding Superb Fairywren, for example, mothers reduce egg investment when breeding in the presence of helpers, but this reduction is obscured by the fact that helpers compensate fully for the undernourishment of hatchlings by assisting in provisioning of young [49]. My findings also support the result of a previous study of *N. vespilloides* [30] showing that in the absence of post-hatching parental care egg size had a strong effect on offspring size, whereas it had no effect on offspring body mass when parents were allowed to care for their larvae. The results of that study as well as the present one may have implications for our understanding of the evolution of egg size, particularly the coevolution of egg size and parental care. In species, where parental care offsets any effects of egg size, selection on egg size may be relaxed leading to smaller eggs. This appears to be supported by some theoretical and empirical evidence from birds suggesting that altricial species, where parents provide elaborate forms of post-hatching care have smaller eggs than precocial species, where parents provide simpler forms of post-hatching care [50–53]. However, despite the fact that egg size did not contribute to final offspring size in the present study, large females laid larger eggs and therefore invested more resources that otherwise could have been allocated to other functions. This finding suggests that there might be other benefits of laying larger eggs than final offspring size. Often, benefits of maternal effects are context-dependent affecting the fitness of phenotypes differently in different environments. For example, there is empirical evidence that egg size raises offspring fitness particularly under harsh environmental conditions, whereas under benign conditions egg size is of less importance [54,55]. This might also apply to burying beetles. Burying beetles do not lay their eggs on the carcass but in the surrounding soil and the hatched larvae have to find their way to the cadaver. Soil humidity might affect the hatching success of large and small eggs differently, or the general locomotion performance of initially large larvae might be superior, thereby facilitating predator avoidance (cf. [54]). Further experiments are required to evaluate possible fitness consequences of egg size in burying beetles.

A final area for consideration is the adaptive significance of maternal effects. Maternal effects can be an effective mechanism to counter variable environments and, therefore, they can be adaptive [8,56]. It is known from several studies that having smaller offspring might not always be a disadvantage, especially when species are confronted with a size–number trade-off and having smaller offspring is just the result of having more offspring [57,58]. Moreover, in specific environments, smaller offspring can have a higher fitness than larger ones [59,60]. However, the size-related postnatal maternal effects shown here in *N. vespilloides* do not appear to be adaptive, but result instead from ‘physiological constraints’. Firstly, small *Nicrophorus* mothers did raise smaller, but not more offspring than large ones. Therefore, the overall efficiency of carrion utilization was lower in small mothers. Secondly, several studies have shown that small beetles are highly disadvantaged in aggressive interactions and therefore have lower chances to secure a carcass for reproduction [25].

In general, maternal effects on progeny traits are common and these can profoundly alter progeny life history. My study demonstrates that in insects with parental care, maternal body size can have prenatal as well as postnatal maternal effects. Moreover, my current research shows that only the postnatal effects persist into adulthood. This finding underscores the importance of disentangling prenatal and postnatal maternal effects and to analyse their relative contribution to final offspring phenotype. Body size is an important issue in evolutionary and life-history theory, and my study provides evidence that there can be transgenerational phenotypic plasticity, with maternal body size determining offspring body size leading to a resemblance between mothers and their offspring irrespective of any direct genetic effects.
